# Epithelial–Mesenchymal Transition in Endometriosis—When Does It Happen?

**DOI:** 10.3390/jcm9061915

**Published:** 2020-06-18

**Authors:** Lutz Konrad, Raimund Dietze, Muhammad A. Riaz, Georgios Scheiner-Bobis, Judith Behnke, Fabian Horné, Alena Hoerscher, Christoph Reising, Ivo Meinhold-Heerlein

**Affiliations:** 1 Institute of Gynecology and Obstetrics, Faculty of Medicine, Justus Liebig University Giessen, 35392 Giessen, Germany; muhammad.a.riaz@gyn.med.uni-giessen.de (M.A.R.); fabian.horne@gmail.com (F.H.); Alena.hoerscher@web.de (A.H.); christoph.reising@gyn.med.uni-giessen.de (C.R.); ivo.meinhold-heerlein@gyn.med.uni-giessen.de (I.M.-H.); 2 Institute of Molecular Biology and Tumor Research (IMT), Philipps University of Marburg, 35037 Marburg, Germany; raimund.dietze@imt.uni-marburg.de; 3 Institute for Veterinary-Physiology and -Biochemistry, School of Veterinary Medicine, Justus-Liebig-University, 35390 Gießen, Germany; Scheiner-bobis@vetmed.uni-giessen.de; 4 Department of General Pediatrics and Neonatalogy, Justus Liebig University Giessen, Universities of Giessen and Marburg Lung Center (UGMLC), Member of the German Center for Lung Research (DZL), 35392 Giessen, Germany; judith.behnke@paediat.med.uni-giessen.de

**Keywords:** endometrium, endometriosis, cell–cell contacts, epithelial–mesenchymal transition, pathogenesis

## Abstract

Epithelial–mesenchymal transition (EMT) is an important process of cell remodeling characterized by the gradual loss of the epithelial phenotype and progressive gain of a mesenchymal phenotype. EMT is not an all-or-nothing process, but instead a transition of epithelial to mesenchymal cells with intermediate cell states. Recently, EMT was described in endometriosis, and many EMT-specific pathways like Twist, Snail, Slug, Zinc finger E-box-binding homeobox 1/2 (ZEB1/2), E/N-cadherin, keratins, and claudins are involved. However, as pointed out in this review, a comparison of the eutopic endometrium of women with and without endometriosis yielded only subtle changes of these EMT markers. Furthermore, only very few alterations in cell–cell contacts could be found but without changes in the epithelial phenotype. This suggests only a partial EMT which is not a prerequisite for the detachment of endometrial cells and, thus, not critical for the first step(s) in the pathogenesis of endometriosis. In contrast, the majority of changes in the EMT-related marker expression were found in the ectopic endometrium, especially in the three endometriotic entities, ovarian, peritoneal, and deep infiltrating endometriosis (DIE), compared with the eutopic endometrium. In this review, we examine the most important EMT pathways described in endometriosis and propose that partial EMT might result from the interaction of endometrial implants with their surrounding microenvironment.

## 1. Endometriosis

Endometriosis is characterized by the presence of endometrial glands and stroma outside of the uterine cavity [1], associated in many cases with pain and/or infertility [2,3]. However, irrespective of location(s) or symptom(s), endometriotic glands almost always have an overtly endometrioid appearance and histologically resemble uterine endometrial glands [1]. Despite this straightforward definition, it is puzzling that endometriotic lesions show so many different facets, such as variations in color, depth of invasion, adhesions, formation of ovarian cysts, and different epithelial-to-stromal cell ratios up to the extreme case of stromal endometriosis [4].

Retrograde menstruation followed by implantation of the endometrial tissue on different surfaces in the pelvic cavity or extra-pelvic sites is generally accepted as the main cause of endometriosis [5]. The prevalence of retrograde menstruation ranges from 76% to 90% but with inconsistent results whether it is higher in women with endometriosis compared to women without endometriosis [6,7,8]. Circulating endometrial cells (CECs) in peritoneal fluid (PF) of 56–79% of women were observed during the follicular or menstrual phase [9,10,11,12]. In a few cases, CECs could also be detected in the PF, but without apparent differences between women with or without endometriosis [12,13]. Interestingly, an increased number of white and red blood cells in the PF are present during the menstrual phase, indicating increased inflammation [13]. In contrast, significantly reduced numbers of uterine natural killer cells were found in the menstrual effluent of patients with endometriosis compared to patients without endometriosis [14].

Worldwide, the prevalence of endometriosis is reported to range from 1% to 10% [15,16,17]. This variation is due to different selections of patient populations and different tools for diagnosing endometriosis with surgical visualization greatly increasing the rates [18]. As long as laparoscopy is the only gold standard for the diagnosis of endometriosis and non-invasive biomarkers are not available [19], the exact determination of the prevalence and incidence of endometriosis will remain unsettled.

In a recent large survey, the anatomical localization of endometriotic lesions was determined in *n* = 1101 patients with 3416 lesions (body mass index (BMI) 21.5; age 33.06 (15–63 years) [20]. The patients showed the majority of lesions in the ovary with 66.9% (56.3% left-sided), in the uterosacral ligament with 45.51% (60.7% left-sided), in the ovarian fossa with 32.15% (67.5% left-sided), in the pouch of Douglas with 29.25%, and in the bladder with 21.25% (57.76% left-sided), while 14.4% had DIE. Another study with *n* = 1500 patients with 10,466 lesions with endometriosis stage IV revealed, for example, 83.91% of lesions in the left broad ligament vs. 71.38% on the right site [21]. Similarly, ovarian endometriosis was evident in 60.21% (left-sided) vs. 49.19% (right-sided). The anatomical distribution and the predominantly left-sided localization were hypothesized to support the menstrual reflux theory of Sampson [21,22].

Peritoneal endometriosis, ovarian endometriosis (endometrioma), and deep infiltrating endometriosis are often classified as three distinct entities, which do not share a common pathogenesis [23]. Ovarian endometriosis, in particular, is thought to be derived from metaplasia [23,24] via transdifferentiation of a committed cell type (e.g., mesothelium) into an alternative cell type (e.g., endometrial epithelium). The occurrence of endometriosis in patients with the Mayer–Rokitansky–Küster–Hauser (MRKH) syndrome who often lack a uterus or endometrium is consistently proposed as evidence for metaplasia as a cause of endometriosis, especially in the ovaries [23,24]. A critical evaluation of the literature about MRKH showed that, whenever biopsies were performed, endometriosis never appeared without a uterus and/or endometrium remnants [25]; however, this does not exclude other hypotheses as possible causes of endometriosis.

In addition to the implantation hypothesis [5] or metaplasia theory [23], alternative hypotheses such as the genetic/epigenetic theory [26], circulating stem/progenitor cells [27], repeated tissue injury and repair (ReTIAR) caused by uterine hyperperistalsis [28], and a fetal [29] or adolescent [30] origin were also suggested. Many arguments were put forward to criticize the implantation hypothesis such as the occurrence of extra-pelvic endometriosis [31]; however, as clearly shown in a recent review [32], Sampson already mentioned the vascular spread. In addition to this recent reappraisal of Sampson’s work, Shakiba et al. [33] showed that patients with hysterectomy combined with laparoscopy experience a dramatically lower recurrence rate of 96.0%, 91.7%, and 91.7% compared to laparoscopy alone with a recurrence rate of 79.0%, 53.3%, and 44.6% after two, five, and seven years, respectively. These data indicate that hysterectomy is associated with a low reoperation rate and that the endometrium is the main reason for endometriosis. Again, this does not exclude other hypotheses in the pathogenesis of endometriosis. Remarkably, recently, it was found that the time to recurrence is independent of the three endometriotic subtypes (ovarian, peritoneal, and DIE) at first surgery [34].

Although the hypothesis of Sampson [5,31] provides a reasonable explanation for the cellular spread, it is still unclear why only 1–10% of women develop endometriosis [15,16,17] despite the high prevalence of retrograde menstruation ranging from 76% to 90% [6,7,8]. Several additional hypotheses such as inflammation, oxidative stress [35], disturbance of the peritoneal barrier [36], and genetic/epigenetic changes [26] were put forward to explain this discrepancy.

It is often described that the eutopic endometrium with and without endometriosis is different [37,38], thus suggesting that the first steps in the pathogenesis of endometriosis might already happen in the endometrium. One of the mechanisms which are suggested to participate at the onset of endometriosis is epithelial–mesenchymal transition (EMT). To the best of our knowledge, the first evidence of EMT in endometriosis was found with endometrial epithelial cells in vitro [39] and later also in vivo alongside the reverse process of mesenchymal–epithelial transition (MET) in human tissue [40].

In this review, we examine the most important EMT pathways involved in human endometriosis, and we only consider pathways investigated in at least three studies. Reviews of other endometrial functions such as decidualization, regeneration, embryo implantation, or adenomyosis in connection with EMT can be found elsewhere [41,42].

## 2. Epithelial–Mesenchymal Transition

EMT is an important conserved mechanism during morphogenesis and organogenesis [43]. In the adult organism, EMT is involved in wound healing, fibrosis, tissue regeneration, inflammation. and cancer metastasis [44,45,46]. EMT programs are classified into three types: (1) type I EMT occurs during embryonic development, (2) type II EMT characterizes wound healing and tissue regeneration, and (3) type III EMT is observed during carcinoma progression [45].

**Figure 1:**
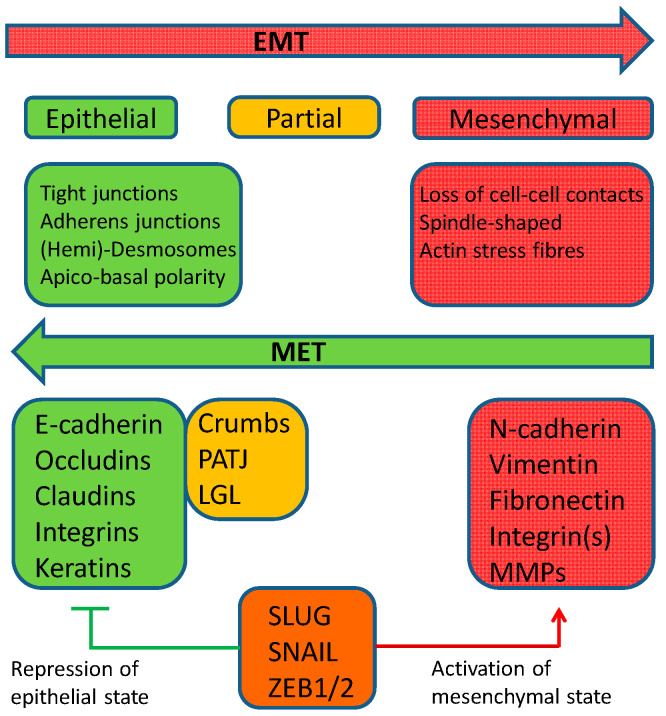
Scheme of the epithelial–mesenchymal transition (EMT)/mesenchymal–epithelial transition (MET) program. Epithelial cells are defined primarily by their cell–cell contacts consisting of tight junctions, adherens junctions, and desmosomes. The contact to the underlying basement membrane is mediated by hemidesmosomes. Proteins associated mainly with the epithelial state modulate cell polarity and cell–cell contact (green box). Induction of EMT by various stimuli results in expression of the EMT transcription factors (TFs) (Zinc finger E-box-binding homeobox (ZEB), Snail, T Twist), red box), which inhibit the expression of “epithelial genes” (green box) and activate the expression of “mesenchymal genes” (red box). The subsequent cellular changes like disassembly of epithelial cell–cell contacts, loss of apical-basal cell polarity, and degradation of the underlying basement membrane are achieved via repression of crumbs, Protein associated with LIN-7 (PALS1)-associated tight junction protein (PATJ), and lethal giant larvae (LGL), which regulate tight junction formation and apical-basal polarity (orange box). During EMT, cells acquire motility and invasive capabilities. MET can reverse EMT and cells revert to an epithelial state. The whole process is neither black and white nor always complete, but it can show intermediate states (orange box). Modified from Dongre and Weinberg [46].

The multi-stage process of EMT comprises the gradual remodeling of epithelial cell architecture and functional capabilities such as loss of epithelial markers resulting in disruption of cell–cell contacts, remodeling of the cytoskeleton, and loss of apical-basal polarity accompanied by the acquisition of mesenchymal markers [43,44,45,46,47]. These changes often cause a mesenchymal phenotype with spindle-like cell shape, as well as increased cell migration, invasion, and cell survival (resistance to anoikis), triggered by several growth factors, cytokines, and numerous transcription factors (Figure 1) [47,48].

The hypothesis that EMT and MET drive the invasion-metastasis cascade was pursued for a long time; however, recent data challenge the role of EMT as a crucial effector of cancer metastasis [47,49,50]. For instance, inhibition of ZEB1/2 by microRNA (miRNA) did not impair metastasis of mouse mammary tumors to the lung [51]. Similarly, loss of either Snail or Twist in pancreatic epithelium suppressed EMT in the primary tumor, and a similar number of metastases in the liver, lungs, and spleen could be observed [52]. Remarkably, both studies reported a higher resistance of cancer cells to chemotherapy because of EMT [51,52]. Another study showed that the EMT transcription factor Twist actually requires intact adherens junctions (E-cadherin) in order to mediate local invasion in breast cancer [53].

Accumulating evidence from the analysis of circulating tumor cells (CTCs) demonstrated the significant heterogeneity of EMT markers supporting the concept of EMT as an important feature of invasive cancer cells [54]. In particular, CTCs with a hybrid epithelial/mesenchymal phenotype showed the highest plasticity to generate an aggressive CTC population which is resistant to chemotherapy and capable of metastatic outgrowth [55]. Thus, it is becoming increasingly clear that EMT and MET are not binary processes, but instead show many hybrids of epithelial/mesenchymal phenotypes with (semi)-stable intermediates [43,44].

Many proteins and signaling pathways were found to be involved in EMT. Loss of E-cadherin, which as a transmembrane protein connects epithelial cells together at adherens junctions, is considered to be a fundamental event in EMT [56]. Several transcription factors (TFs) repress E-cadherin directly or indirectly such as Snail1, Slug, ZEB1/2, Twist, Goosecoid, and fork-head box protein C2 (FOXC2) [57]. These factors may regulate each other in a hierarchical pattern where Snail1 and Slug are initially induced, leading to the activation of the above-mentioned factors [57]. Interestingly, they also modulate expression of claudins and desmosomes, thus facilitating EMT. For example, Slug and Snail repress claudin-1 messenger RNA (mRNA) and protein expression in vitro [58] and Slug triggers desmosomal disruption, the first and necessary phase of EMT [59]. Although Slug induced ZEB1 and fibronectin expression [60], it did not trigger the second phase consisting of cell motility, repression of cytokeratin expression, and activation of vimentin expression [59].

## 3. Mesenchymal–Epithelial Transition (MET)

MET, the reverse process of EMT, is less well characterized, and its role in metastatic outgrowth is still unresolved [60]. Cells that undergo EMT proliferate less and, thus, cannot colonize the metastatic site. Up to date, the carcinoembryonic antigen-related cell adhesion molecule 5 (CEACAM5) was identified so far as a possible regulator of MET and metastatic colonization [61]. Additionally, the transcription factors ovo-like transcriptional repressor (Ovol1/2) and grainyhead-like (GRHL2), also known as phenotypic stability factors, were reported to regulate MET via ZEB1 [62,63]. However, neither Ovol1/2 nor GRHL2 were analyzed in endometriosis, and the involvement of MET in peritoneal endometriosis was only described in one report [40].

## 4. Materials and Methods

In PubMed, we searched for articles describing an association between EMT and endometriosis (Figure S1, Supplementary Materials). Thus, we performed a systematic retrospective literature review. We looked for the following keywords: E/N-cadherin, Snail1, Slug (also known as Snail2), Twist, Ovol1/2, GRHL2, claudin(s), occludin, integrin(s), keratin(s), vimentin, transforming growth factors-beta (TGF-βs), epidermal growth factor (EGF), hepatocyte growth factor (HGF), phosphatidylinositol 3′ kinase/Ak strain transforming (PI3K/Akt), mitogen-activated protein kinase (MAPK), Wingless Int-1 (Wnt)/β-catenin, notch, estrogen receptor-alpha (ER-α), Crumbs, Hedgehog signaling pathway, nuclear factor kappa B (NF-κB), activating transcription factor 2 (ATF2), Protein associated with LIN-7 (PALS1)-associated tight junction protein (PATJ), lethal giant larvae (LGL), and fibronectin. Only manuscripts showing immunohistochemistry of eutopic and ectopic endometrium together with a scoring system and at least three studies published were included in this review. Most often, the scoring system of − (0), + (1), ++ (2), and +++ (3), but very rarely the Histological Score (HSCORE), was used for evaluation of immunohistochemical staining. Information about hormonal contraception is not included because no studies compared it with no treatment. Sometimes, we had to deduce the immunohistochemical values from the graphs. In these cases, we could only give approximate values, and we calculated the *p*-values by ourselves (both marked by brackets). Non-parametric comparisons between two groups were done with the Mann-Whitney test and those between three or more groups were done with the Kruskal-Wallis test.

## 5. The Role of EMT in Endometriosis

In the last few years, numerous manuscripts were published suggesting the involvement of EMT in the pathogenesis of endometriosis [41,42]. In this section, we examine the most important EMT-related factors involved in endometriosis.

### 5.1. Epithelial Markers

#### 5.1.1. E-Cadherin (Cadherin-1) in Endometriosis

Loss of cell–cell adhesion mediated by or associated with decreased E-cadherin protein abundance was shown to transform cells to spindle-like mesenchymal cells and enhance their migratory and invasive behavior [64,65].

E-cadherin was the most frequently analyzed EMT marker in endometriosis. In 13 out of 16 manuscripts, information about the cycle phases in the normal endometrium was provided; however, cycle-specific differences were not analyzed (Table 1). Only one study reported no cycle-specific differences [66], but it was not included in Table 1, because no comparison between eutopic and ectopic endometrium was provided. In seven out of 16 studies, a comparison of eutopic endometrium in cases with and without endometriosis showed no significant differences, except for two reports claiming a significant reduction [67,68] (Table 1). Comparison of peritoneal and/or ovarian endometriosis to eutopic endometrium revealed in nearly all studies a reduced E-cadherin expression and a significant difference between ectopic endometrium (especially peritoneal and ovarian) and eutopic endometrium (Table 1). In only one manuscript, the three different endometriotic entities were evaluated, and they demonstrated a higher E-cadherin protein expression in DIE compared to ovarian and peritoneal endometriosis, which was even higher compared to eutopic menstrual endometrium [40].

##### 5.1.2. β-Catenin in Endometriosis

Wnt/β-catenin signaling could inhibit E-cadherin expression through EMT transcription factors (EMT-TFs) Twist and Slug [57]. Thus, we analyzed β-catenin expression in eutopic and ectopic endometrium, and we could only identify four reports (Table 2). Unfortunately, no information about the cycle-dependency was published. In two studies, a comparison of eutopic endometrium with and without endometriosis demonstrated no differences in β-catenin expression [73,80]. In three reports, a significantly lower β-catenin expression in ectopic compared to eutopic endometrium was found (Table 2). In contrast, one study reported an even higher β-catenin expression in ectopic endometrium [80].

Similarly to E-cadherin, the β-catenin expression pattern in eutopic endometrium with and without endometriosis did not reveal too many differences. Although E-cadherin and β-catenin expression is decreased in ectopic endometrium, it remains to be elucidated whether endometrial epithelial cell–cell contacts are also impaired.

#### 5.1.3. Cell–Cell Contacts (Claudins) in Endometriosis

As mentioned above, EMT-TFs also regulate claudin expression. Since the loss of cell–cell contacts is a prerequisite for the loss of the epithelial phenotype [45,46], we address in this section whether claudin expression is altered in endometriosis.

Human endometrium expresses claudins-1–5, 7, and 10 [82]. Up to now, only four studies were published regarding claudin expression in endometriosis with their findings summarized in Table 3. In contrast to one study showing cycle-dependent differences of mRNA expression of claudins [80], three studies found no differences in expression of claudins-2–4 and 11 [83,84,85,86]. Again, no significant differences between the eutopic endometrium of women with and without endometriosis could be shown for claudins-2–4, 7, and 11 (Table 3).

In contrast to claudins-2, 5, 7, and 11, which are not significantly different in the ectopic endometrium compared to eutopic endometrium [83,84,85,86], claudin-1 and claudin-4 are significantly different, showing a decreased protein presence in peritoneal endometriosis [83]. Furthermore, claudin-4 was found to also be different in ovarian endometriosis [84,85] (Table 3). Instead, data for claudin-3 are controversial; whereas Pan et al. [84] found a reduced protein presence in ovarian endometriosis, Hoerscher et al. [86] did not identify any changes, but in contrast identified claudin-3 expression in nearly all glands in the ectopic endometrium.

Although, for claudin-11, no differences in the HSCORE could be found between eutopic and ectopic endometrium, a significant switch of claudin-11 localization to a basal or basolateral localization in ovarian, peritoneal, and DIE could be observed [85]. In particular, in ovarian endometriosis, a shift to a more basal or basolateral localization was prominent compared to a preferential apicolateral localization in eutopic endometrium [85].

Taken together, the analysis of claudins in endometriosis showed only the loss of few claudins, but not of all, especially in the eutopic endometrium.

### 5.2. Mesenchymal EMT Markers

#### 5.2.1. Snail and Slug (Snail2) in Endometriosis

We identified four manuscripts showing Snail localization in eutopic and ectopic endometrium (Table 4). Only one study [66], reporting no differences in cycle-dependent expression, was not included, because no comparison between eutopic and ectopic endometrium was provided. In one study, a comparison of eutopic endometrium with and without endometriosis revealed an identical percentage of Snail-positive epithelial cells [80], again questioning EMT at the endometrial stage. In three out of four studies, Snail protein presence was higher in the ectopic endometrium, especially in ovarian endometriosis, as reported in two studies [79,80] (Table 4).

For Slug, we could identify only one report, demonstrating no increase [77].

#### 5.2.2. ZEB1 in Endometriosis

Localization of ZEB1 was presented in only three studies (Table 5). In one study, no cycle-dependent changes were reported, as well as no differences in the eutopic endometrium in cases with and without endometriosis [87]. In contrast, Wu et al. [67] found an eight-fold increase in ZEB1 abundance in the eutopic endometrium of cases with endometriosis. However, all three reports agree that ZEB1 is significantly upregulated in the ectopic endometrium, especially in DIE [87,88] (Table 5).

#### 5.2.3. Twist in Endometriosis

Although no cycle-dependency was analyzed, the comparison between the eutopic endometrium of cases with and without endometriosis showed either an increase [68] or a reduction [90] in Twist protein expression in cases with endometriosis (Table 5). All studies reported an increased Twist expression in ectopic compared to eutopic endometrium [68,77,89,90].

#### 5.2.4. Vimentin in Endometriosis

All in all, we could identify seven studies with vimentin, showing somewhat heterogeneous results (Table 6). In contrast to a significant cycle-dependent decrease in the secretory phase [91,92], one report found no differences [88]. The comparison between the eutopic endometrium of cases with and without endometriosis was either significant [67] or not significant [78,88]. Similarly controversial are the comparisons of ectopic with eutopic endometrium; three reports found a decrease in eutopic endometrium [78,88,92], and one report found a decrease and an increase in eutopic endometrium [40]. Whereas four reports identified a significant vimentin increase in ovarian endometriosis [67,78,79], two studies reported a decrease [88,92].

#### 5.2.5. N-Cadherin (Cadherin-2) in Endometriosis

Decreased expression of E-cadherin is often associated with an increased expression of N-cadherin [93]. In one report, a significantly reduced N-cadherin expression in the secretory compared to the proliferative phase was reported [94] (Table 7), whereas Qi et al. [66] identified no differences. Two studies found no differences in the eutopic endometrium of cases with endometriosis compared to cases without endometriosis [40,78]. The majority of studies presented an increased N-cadherin expression in the ectopic compared to the eutopic endometrium [40,68,77,78]. In contrast, one report could not identify any changes [75].

Remarkably, the mesenchymal marker N-cadherin was also not differentially expressed in the eutopic endometrium of cases with and without endometriosis.

## 6. EMT in Endometriosis—When Does It Happen?

### 6.1. Summary of the Epithelial EMT Markers in Endometriosis

We found that the cycles (25/28 = 89.3%) and hormonal treatments (13/28 = 46.4%) are often but not always reported. Furthermore, in eight reports [84,85,86,87,88,91,92,94], proliferative and secretory phases were compared. Thus, this review has some limitations; however, despite these shortcomings, several conclusions can be clearly drawn from the present investigation.

Taken together, in most studies (14/16 = 87.5%), no significant differences in E-cadherin (6/8), β-catenin (2/2) and claudins (6/6) in the eutopic endometrium with or without endometriosis could be identified (Table 1, Table 2 and Table 3). Thus, we can conclude with a high probability that the epithelial phenotype is retained in the endometrium of cases with endometriosis and, therefore, EMT is not a prerequisite for the dissemination of endometrial cells. In contrast, the comparison of the ectopic with the eutopic endometrium paints a different picture. Many studies showed a decreased expression of E-cadherin (10/12 = 83.3%) and β-catenin (4/4 = 100%) in the ectopic compared to the eutopic endometrium. Despite this clearly reduced expression, the claudin-dependent tight junctions are mostly retained in the ectopic endometrium (7/11 = 63.6%), as well as in the three different entities (Table 3). In contrast, the three endometriotic entities showed a different pattern of E-cadherin expression with a higher abundance in DIE and black peritoneal lesions compared to ovarian and peritoneal lesions (Table 1).

From all the above, we conclude that cell–cell contacts remain nearly intact in the ectopic endometrium despite the loss in expression of E-cadherin and β-catenin. This suggests that at least a partial EMT with retained cell–cell contacts takes place in the ectopic endometrium, possibly after implantation. However, this partial EMT does not include any loss of the epithelial phenotype. Our conclusions are new and clearly different to the observations with cancer cells, where EMT is considered to be a prerequisite of dissemination [45].

Up to date, a detailed molecular signature and morphological features as robust hallmarks to define partial EMT remain to be identified, and it is still unclear whether these partial EMT states are metastable or transient snapshots [95]. Furthermore, the co-expression of epithelial and mesenchymal markers in the same cell or in a subset of cells remains to be clarified. In the case of a partial EMT in endometriosis, we observed a strong expression of cell–cell contacts in nearly all endometrial and endometriotic glands, as well as in nearly all epithelial cells [85,86,88], thus suggesting that the partial EMT might occur in the same cell(s).

Further in line with our assumptions of a partial EMT in the pathogenesis of endometriosis are recent observations on somatic mutations in the eutopic and ectopic endometrium. The findings that endometrial stromata do not share mutations with the epithelium neither in the eutopic or in the ectopic endometrium [96] clearly suggest that endometrial epithelial cells do not transform into stromal cells. Our hypothesis of a partial EMT is in agreement with these observations, because we demonstrate that epithelial cells do not lose their epithelial phenotype and show only a partial gain of mesenchymal marker expression. Recently, it was shown that the clonal expansion of endometriotic lesions is driven by mutations in cancer-associated genes with a higher distribution compared to normal endometrial epithelium [97]. The clonality of endometriotic lesions is in accordance with our assumption that epithelial cells do not transform into stromal cells. Similarly, the higher distribution of mutations in cancer-associated genes in endometriotic lesions [97] is comparable to our observation that most of the changes in EMT markers might occur after implantation.

From all these assumptions, it is now clear why we did not include in this review comparative studies involving endometrial and endometriotic cells. Since most of the EMT changes occur after implantation, the comparison between these two distinct cell types would not have provided any information about the first steps in the pathogenesis of endometriosis.

### 6.2. Summary of the Mesenchymal EMT Markers in Endometriosis

We found only in very few reports a slight gain in expression of mesenchymal EMT markers in the eutopic endometrium with and without endometriosis (2/11 = 18%, Table 4, Table 5, Table 6 and Table 7). In contrast, the mesenchymal markers in the ectopic endometrium compared to the eutopic endometrium demonstrated an increase in the majority of studies (22/24 = 92%; Table 4, Table 5, Table 6 and Table 7), which is much higher compared to 18% in the eutopic endometrium. Among the endometriotic entities, a remarkable difference could be identified in 4/4 (100%) studies.

**Figure 2:**
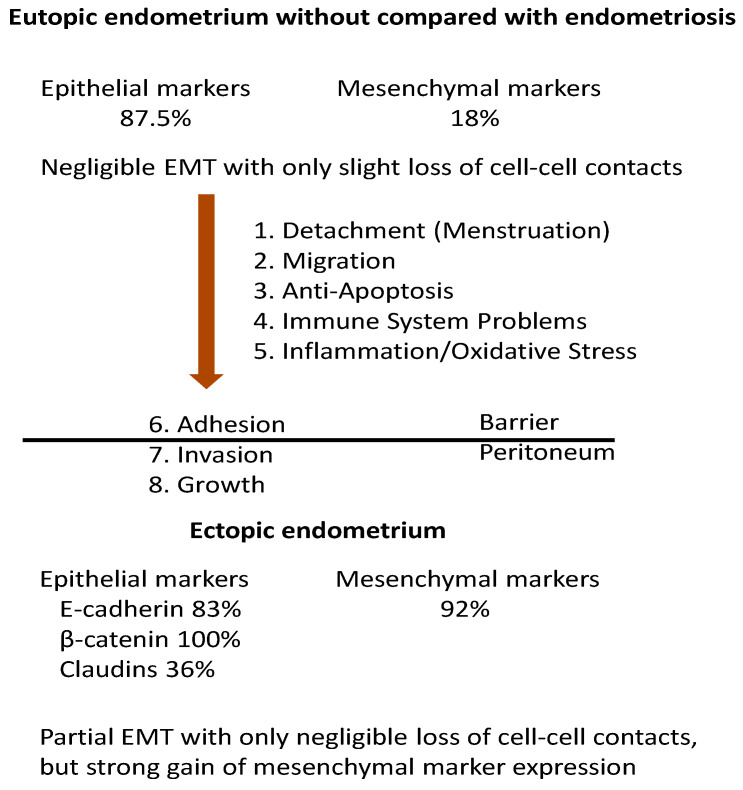
EMT in endometriosis. At the level of the endometrium, the epithelial EMT markers showed no differences in 87.5% of the studies, whereas the mesenchymal markers showed differences only in 18% of the studies when the eutopic endometrium of patients with and without endometriosis was compared. This suggests a partial EMT with only negligible loss of cell–cell contacts. A decreased protein expression of E-cadherin was found in 83.3% of the studies, but differences in the eutopic endometrium and the ectopic endometrium could be shown in 36.4% of the studies with claudins. The mesenchymal markers in the ectopic endometrium were different in 92% of the studies compared to the eutopic endometrium. Thus, we suggest that, after implantation, EMT was still partial with only a negligible loss of cell–cell contacts but with a strong gain in the expression of mesenchymal markers. In summary, EMT in endometriosis occurs mainly after implantation.

Thus, the decreased expression of epithelial EMT markers is inversely correlated to the increased expression of mesenchymal EMT markers in the ectopic endometrium. This strengthens our previous assumptions that most of the EMT-like changes occur after implantation (Figure 2).

### 6.3. Perspectives and Clinical Relevance of EMT in Endometriosis

According to our assumptions, we predict that the analysis of CECs from patients with and without endometriosis will not reveal too many differences with respect to EMT marker expression, although this analysis is necessary. The distinct EMT patterns in the three endometriotic entities suggest that there might be differences such as inflammation or other factors in the distinct host tissues of women with endometriosis compared to healthy women, thereby causing EMT after implantation. Various aspects of the different microenvironments on the lesions [98,99], as well as the different rates of somatic mutations or genetic/epigenetic incidents of the endometriotic lesions compared to the eutopic endometrium [26,96], seem to be mainly responsible for their heterogeneity and, thus, for the clinical symptoms of endometriosis. In other words, we believe that we should focus more on characterization of the “soil” instead of the “seed” to treat women susceptible for endometriosis before they get the disease. The implications for the clinicians are obvious; endometriosis should be better prevented by the use of mechanical barriers such as for example levonorgestrel-releasing intrauterine devices (LNG-IUDs), which are also helpful in reduction of recurrence [100]. Furthermore, the total ablation of the endometrium in women without the wish to conceive should be used more often [32]. Furthermore, reduction of repetitive stress, reduction of microtrauma, and prevention of peritoneal inflammation may be useful [26].

## 7. Conclusions

The vast majority of studies showed only very little evidence of EMT at the level of the endometrium (Table 1, Table 2, Table 3, Table 4, Table 5, Table 6 and Table 7); thus, we suggest that EMT is not involved in the early steps of endometriosis such as loss of cell–cell contacts followed by dissemination of endometrial cells. While tumor cells need strategies such as EMT in order to escape tight epithelial structures to metastasize, the detachment of endometrial cells is facilitated by the physiological breakdown during menstruation. Indeed, endometrial single cells, clumps of cells, cells with stem cell-like features, and gland-like structures of epithelial and stromal/mesenchymal phenotype, were observed in peripheral blood and menstrual effluent [101,102,103].

Currently, we cannot answer the question whether EMT occurs during dissemination of endometrial cells. However, we suppose that not too many differences will be observed, because, until now, the few studies on CECs observed only negligible qualitative differences between women with or without endometriosis [104,105]. Although two reports claimed to have identified more stromal [106] or more epithelial and mesenchymal CECs [107] in the peripheral blood, no differences were observed when CECs were examined in the PF of women with or without endometriosis [12,13]. Remarkably, CECs can induce EMT in mesothelial cells [108,109], thus highlighting the importance of the mesothelial barrier in the pathogenesis of endometriosis [36,110]. Therefore, we suggest that future studies should focus on the characterization of CECs with mesenchymal and epithelial markers, but more importantly on an analysis of differences in the “soil” of women with and without endometriosis.

Most studies showed a clear expression of EMT markers in the ectopic endometrium. The differential expression of EMT markers in ovarian, peritoneal, and deep infiltrating endometriosis is limited by the fact that all three entities are rarely investigated together. In contrast, ectopic endometrial epithelial cells retain their expression of various epithelial markers such as most claudins (Table 3) and cytokeratins [88,92], thereby not losing their epithelial phenotype. As shown with an in vitro invasion assay, the integrity of the tissue architecture (epithelial and stromal unit) was pivotal for the ability of the endometrium to infiltrate and to form lesions [111]. Subtle changes in the expression of cell–cell contacts and more pronounced changes in the presence of EMT markers suggest only a partial EMT occurring in the ectopic endometrium. This is further emphasized by the analysis of somatic mutations which clearly excluded a complete transition of endometrial epithelial to stromal cells [96], but not a partial EMT. We are inclined to assume that the partial EMT, which seems to be distinctly different in the three endometriotic entities, is a result of the interaction of the endometriotic implant with the different environments (e.g., peritoneal fluid, ovarian hormones, or different tissue environment) as suggested by Koninckx et al. [98,99]. Furthermore, EMT of the ectopic endometrium might also be partially responsible at least for the diversity in the three entities. Similarly, Feider et al. [112] and Guo [113] also mentioned that the differences between eutopic and ectopic endometrium might be due to their immediate environment rather than inherent differences between eutopic and ectopic endometrium. This does not exclude the possibility of inherited (somatic) mutations with additional genetic and epigenetic incidents in cystic ovarian endometriosis and DIE, as proposed by the genetic/epigenetic theory [26]; however, it remains unclear whether these changes occur preferentially in the endometrial epithelial or stromal cells.

Taken together (Figure 2), we suggest the following:

- EMT in endometriosis is not involved as a main factor in the dissemination of endometrial cells;
- Only a partial EMT takes place after implantation with no significant loss of cell–cell contacts and no loss of the epithelial phenotype;
- The partial EMT results from the different microenvironments at the distinct integration sites of the endometrial implants;
- This is a new type of EMT which we term type IV.

## Figures and Tables

**Table 1:** Immunohistochemistry of E-cadherin in eutopic and ectopic endometrium.

Eutopic Endometrium						Ectopic Endometrium (Entities)					
Refs	All (*n*)	Without EM	With EM	Scores	*p*-Value	All (*n*)	Ov. EM	PE	DIE	Scores	*p*-Value
[69]	16	8	8	100%	n.d.	16	n.d.	8	n.d.	−16.70%	n.d.
[70]	19	n.sp.	n.sp.	2.2 P/2.8 S	n.d.	10	n.sp.	n.sp.	n.sp.	2.7 P/3.2 S	n.d.
[71]	26	n.sp.	n.sp.	99%	n.d.	9	n.sp.	n.sp.	n.sp.	33%	n.d.
[72]	18	n.d.	18	100%	n.d.	18	n.d.	18	n.d.	89%	n.d.
[73]	49	25	24	2.24 ^a^/1.83 ^b^	(n.s.)	21	n.d.	21	n.d.	(0.52 ^c^)	(<0.001 ^a,b/c^)
[74]	7	n.sp.	n.sp.	(1.71 ^a^)	n.d.	11	11	n.d.	n.d.	(0.82 ^b^)	(0.0025 ^a/b^)
[75]	15	15	n.d.	92% ^a^	n.d.	23	n.d.	23	n.d.	50% ^b^	<0.01 ^a/b^
[76]	10	n.sp.	n.sp	91%	n.d.	15	n.sp.	n.sp.	n.sp.	85%	n.s.
[40]	32	14	18	11.2 ^a^	n.s.	199	55	30 red, 46 black	68	3.3 ^b^/2.9 red ^c^/19.4 black ^d^/20.3 ^e^	0.03 ^a/d^
											0.005 ^a/e^
											0.0001 ^b,c/d^
											0.0001 ^b,c/e^
[77]	12	12	n.d.	2.58	n.d.	25	9	9	9	2.76 all three	n.s.
[78]	41	20	21	8.35 ^a^/7.24 ^b^	n.s.	21	21	n.d.	n.d.	5.14 ^c^	0.0325 ^a/c^
											0.0089 ^b/c^
											<0.01 ^a,b/c^
[79]	40	40	n.d.	(~0.35)	n.d.	40	40	n.d.	n.d.	(~0.05)	≤0.05
[67]	23	12	11	(~38 ^a^/~20 ^b^)	≤0.05 ^a/b^	11	11	n.d.	n.d.	(~18 **)	<0.01 ^a/c^
[68]	60	30	30	(1.83 ^a^/0.73 ^b^)	(0.001 ^a/b^)	30	30	n.d.	n.d.	(0.27 ^c^)	≤0.05 ^a,b/c^
[80]	42	21	21	6.38 ^a^/7.43 ^b^	n.s.	21	21	n.d.	n.d.	3.86 ^c^	≤0.05 ^a/c^
											<0.01 ^b/c^
[81]	110	50	60	(~7.3 ^a^/7.2 ^b^)	n.s.	65	65	n.d.	n.d.	(~5.0 ^c^)	≤0.05 ^a,b/c^

Refs, references; % denotes the percentage of stained samples; EM, endometriosis; Ov. EM, ovarian endometriosis; PE, peritoneal endometriosis; DIE, deep infiltrating endometriosis; n.d., not done; n.sp., not specified; n.s., not significant; P, proliferative; S, secretory. Superscript letters denote the statistical comparisons.

**Table 2:** Immunohistochemistry of β-catenin in eutopic and ectopic endometrium.

Eutopic Endometrium						Ectopic Endometrium (Entities)					
Refs	All (*n*)	Without EM	With EM	Scores	*p*-Value	All (*n*)	Ov. EM	PE	DIE	Scores	*p*-Value
[73]	49	25	24	1.8 ^a^/1.58 ^b^	(n.s.)	21	n.d.	21	n.d.	(0.76 ^c^)	(≤0.05 ^b/c^)
											(<0.001 ^a/c^)
[74]	7	n.sp.	n.sp.	(1.71 ^a^)	n.d.	11	11	n.d.	n.d.	(0.91 ^b^)	(0.0095 ^a/b^)
[76]	10	n.sp.	n.sp	91%	n.d.	15	n.sp.	n.sp.	n.sp.	72%	0.002
[80]	42	21	21	14.3%/23.8%	n.s.	21	21	n.d.	n.d.	61.9%	n.d. ≤0.05 ^b/c^
				2.57 ^a^/3.62 ^b^						7.14	<0.01 ^a/c^

Refs, references; % denotes the percentage of stained patients (p); EM, endometriosis; Ov. EM, ovarian endometriosis; PE, peritoneal endometriosis; DIE, deep infiltrating endometriosis; n.d., not done; n.sp., not specified; n.s., not significant. Superscript letters denote the statistical comparisons.

**Table 3:** Immunohistochemistry of claudins in eutopic and ectopic endometrium.

Eutopic Endometrium (Phases)						Ectopic Endometrium (Entities)					
Refs	All (*n*)	Without EM	With EM	Scores	*p*-Value	All (*n*)	Ov. EM	PE	DIE	Scores	*p*-Value
Claudin-1											
[83]	22	13	9	(2.05 ^a^ both)	n.d.	17	n.d.	17	n.d.	(1.47 ^b^)	(0.0187 ^a/b^)
Claudin-2											
[86]	26 P, S	12	14	165 P/178 S 176/189	n.s. n.s.	19	6	6	7	179/220/209	n.s.
Claudin-3											
[83]	22	13	9	(2.27 both)	n.d.	(20)	n.d.	(20)	n.d.	(2.0)	n.s.
[84]	62 P, S	35	27	0.53 P/0.6 S0.57 ^a^/0.59 ^b^	n.s. n.s.	35	35	n.d.	n.d.	0.17 ^c^	≤0.05 ^a/c^ ≤0.05 ^b/c^
[86]	51 P, S	19	32	268 P/253 S261/270	n.s. n.s.	62	20	17	25	266/263/286	n.s.
Claudin-4											
[83]	20	n.sp.	n.sp.	(2.3 ^a^ all)	n.d.	20	n.d.	20	n.d.	(1.4)	(0.0031 ^b^)
[84]	62 P, S	35	27	0.6 P/0.85 S 0.74 ^a^/0.7 ^b^	n.s.	35	35	n.d.	n.d.	0.20 ^c^	≤0.05 ^a/c^ ≤0.05 ^b/c^
Claudin-5											
[83]	21	n.sp.	n.sp.	(1.9 all)	n.d.	19	n.d.	19	n.d.	(1.42)	(n.s.)
Claudin-7											
[83]	19	n.sp	n.sp	(2.21 all)	n.d.	21	n.d.	21	n.d.	(1.81)	(n.s.)
[85]	49 P, S	18	31	232 P/249 S 241/237	n.s. n.s.	62	20	17	25	235/278/257	n.s.
Claudin-11											
[85]	49 P, S	18	31	185 P/187 S 186/169	n.s.	62	20	17	25	125/182/159	n.s.

Refs, references; EM, endometriosis; Ov. EM, ovarian endometriosis; PE, peritoneal endometriosis; DIE, deep infiltrating endometriosis; n.d., not done; n. sp, not specified; n.s., not significant; P, proliferative; S, secretory. Superscript letters denote the statistical comparisons.

**Table 4:** Immunohistochemistry of Snail in eutopic and ectopic endometrium.

Eutopic Endometrium (Phases)						Ectopic Endometrium					
Refs	All (*n*)	Without EM	With EM	Scores	*p*-Value	All (*n*)	Ov. EM	PE	DIE	Scores	*p*-Value
[77]	12	12	n.d.	1.58 ^a^	n.d.	24	n.sp.	n.sp.	n.sp.	1.96 ^b^	0.027 ^a/b^
[87]	10	10	n.d.	100% p	n.d.	7	6 lesions	n.d.	5 lesions	100% p	n.d.
[79]	40	40	n.d.	(~0.04)	n.d.	40	40	n.d.	n.d.	(~0.52)	<0.001
[80]	42	21	21	19% ^a^/19% ^b^ 3.43 ^d^/2.71 ^e^	n.s.	21	21	n.d.	n.d.	57.1% ^c^ 5.86 ^f^	≤0.05 ^a,b/c^ ≤0.01 ^e/f^

Refs, references; % denotes the percentage of stained samples; EM, endometriosis; Ov. EM, ovarian endometriosis; PE, peritoneal endometriosis; DIE, deep infiltrating endometriosis; n.d., not done; n. sp, not specified; n.s., not significant. Superscript letters denote the statistical comparisons.

**Table 5:** Immunohistochemistry of ZEB1 and Twist in eutopic and ectopic endometrium.

Eutopic Endometrium						Ectopic Endometrium (Entities)					
Refs	All (*n*)	Without EM	With EM	Scores	*p*-Value	All (*n*)	Ov. EM	PE	DIE	Scores	*p*-Value
ZEB1											
[87]	10	10	n.d.	0% ^a^ s	n.d.	7	6 lesions	n.d.	5 lesions	45% ^b^ s	0.0039 ^a/b^
[67]	23	12	11	~(25 ^a^/200)	<0.001	11	11	n.d.	n.d.	(~125 ^b^)	≤0.05 ^a/b^
[88]	50	20	30	104/71 84 ^a^ both	n.s.	75	30 lesions	21 lesions	27 lesions	145/138/202 ^b^	<0.001 ^a/b^
Twist											
[77]	13	13	n.d.	1.31 ^a^	n.d.	26	9	9	9	2.42 ^b^ all	<0.001 ^a/b^
[89]	119	50	69	(~17% with EM)	n.d.	86	n.sp.	n.sp.	n.sp.	(~50%)	<0.001
[90]	119	50	69	(~3.0/~1.8 ^a^)	0.001	90	n.sp.	n.sp.	n.sp.	(~3.0 ^b^)	<0.001 ^a/b^
[68]	60	30 Infertile	30	(0.17 ^a^/0.47 ^b^)	n.s.	30	30	n.d.	n.d.	(1.67 ^c^)	(<0.001 ^a,b/c^)

ZEB1, Zinc finger E-box binding homeobox; Refs, references; % denotes the percentage of stained samples (s), EM, endometriosis; Ov. EM, ovarian endometriosis; PE, peritoneal endometriosis; DIE, deep infiltrating endometriosis; n.d., not done; n. sp, not specified; Superscript letters denote the statistical comparisons.

**Table 6:** Immunohistochemistry of vimentin in eutopic and ectopic endometrium.

Eutopic Endometrium (Phases)						Ectopic Endometrium (Entities)					
Refs	All (*n*)	Without EM	with EM	Scores	*p*-Value	All (*n*)	Ov. EM	PE	DIE	Scores	*p*-Value
[91]	29 P, S	29	n.d.	188 ^a^ P, 140 ES 93 ^b^ LS	<0.01 ^a/b^	31	n.d.	31 black	n.d.	63 P, 80 ES, 55 LS	n.s.
[92]	20	n.d.	20	342 ^a^ P, 252 ^b^ S	<0.01 ^a/b^	20	20	n.d.	n.d.	185 ^c^ P, 130 ^d^ S	≤0.05 ^c/d^
[40]	32	14	18	10.5 ^a^	n.d.	199	55	30 red 46 black	68	1.5 ^b^/34.3 red ^c^/63.4 black ^d^/20.5 ^e^	<0.0001 ^a/c,d,e^ <0.0001 ^b/c,d,e^
[78]	41	20	21	10% ^a^/23.8% ^b^	n.s.	21	21	n.d.	n.d.	61.9% ^c^	0.0278 ^b/c^ 0.0009 ^a/c^
[79]	40	40	n.d.	(~0.23)	n.d.	40	40	n.d.	n.d.	(~0.44)	<0.01
[67]	23	12	11	(~20 ^a^/~90)	0.01	11	11	n.d.	n.d.	(~95 ^b^)	<0.01 ^a/b^
[88]	50	20	30	65/76 72 ^a^ both	n.s.	78	30	27	21	15 ^b^/46 ^c^/94	<0.01 ^a/b^ <0.001 ^b/c^

Refs, references; % denotes the percentage of stained samples; EM, endometriosis; Ov. EM., ovarian endometriosis; PE, peritoneal endometriosis; DIE, deep infiltrating endometriosis; n.d., not done; n. sp., not specified; n.s., not significant; P, proliferative; S, secretory; ES, early secretory; LS, late secretory. Superscript letters denote the statistical comparisons.

**Table 7:** Immunohistochemistry of N-cadherin in eutopic and ectopic endometrium.

Eutopic Endometrium						Ectopic Endometrium (Entities)					
Refs	All (*n*)	Without EM	With EM	Scores	*p*-Value	All (*n*)	Ov. EM	PE	DIE	Scores	*p*-Value
[94]	26	26	n.d.	4.2 ^a^ P/1.5 ^b^ S	<0.01 ^a/b^	34	n.d.	13	21	1.7 ^c^/2.9 ^d^	≤0.05 ^a/c^
[68]	60	30	30	10%/30% 0.1 ^a^/0.3 ^b^	n.d.	30	30	n.d.	n.d.	60% 0.77 ^c^	≤0.05 ^b/c^ <0.001 ^a/c^
[78]	40	20	21	5% ^a^/9.5% ^b^ 1.75 ^a^/1.38 ^b^	n.s. ˃0.05 ^a/b^	21	21	n.d.	n.d.	38.1% 3.67 ^c^	0.02 ^a/c^ <0.05 ^a,b/c^
[77]	13	13	n.d.	(1.85 ^a^)	n.d.	27	9	9	9	(2.54 ^b^)	(0.005 ^a/b^)
[40]	32	14	18	0/0 ^a^	n.s.	199	55 ^b^	30 ^c^ red, 46 ^d^ black	68 ^e^	1.2 ^b^/3.9 ^c^/1.7 ^d^/1.3 ^e^	0.0001 ^a/c^ 0.0006 ^c/d^ 0.006 ^a/e^
[75]	15	15	n.d.	100%/93%	n.d.	23	n.d.	23	n.d.	100%/89%	n.s.

Refs, references; % denotes the percentage of stained samples; EM, endometriosis; Ov. EM, ovarian endometriosis; PE, peritoneal endometriosis; DIE, deep infiltrating endometriosis; n.d., not done; n.sp., not specified; n.s., not significant; P, proliferative; S, secretory. Superscript letters denote the statistical comparisons.

## Supplementary Materials

The following are available online at https://www.mdpi.com/2077-0383/9/6/1915/s1, Figure S1: PRISMA flowchart of literature search and data selection.
