# Supplementary figures and images for: Epithelial–Mesenchymal Transition in Endometriosis—When Does It Happen?
Source: J Clin Med. 2020 Jun 18;9(6):1915. doi: 10.3390/jcm9061915 (PMC7357060; doi:10.3390/jcm9061915)

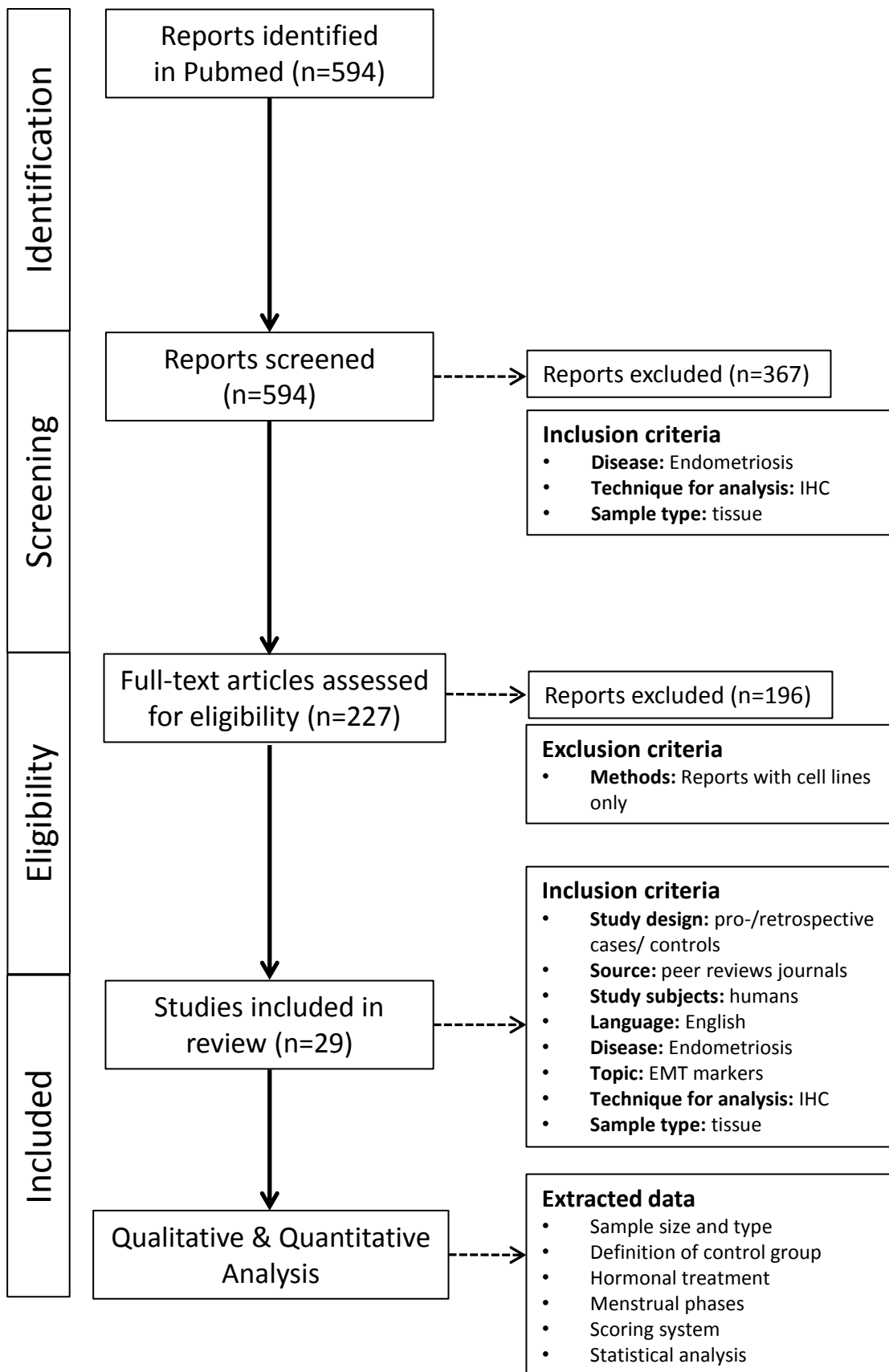

Figure S1

Supplement: Supplementary file 1 [file jcm-09-01915-s001.pdf]
